# Follicle-Stimulating Hormone Biological Products: Does Potency Predict Clinical Efficacy?

**DOI:** 10.3390/ijms24109020

**Published:** 2023-05-19

**Authors:** Monica Lispi, Peter Humaidan, George R. Bousfield, Thomas D’Hooghe, Alfredo Ulloa-Aguirre

**Affiliations:** 1 Merck Healthcare KGaA, 64293 Darmstadt, Germany; thomas.dhooghe@merckgroup.com; 2 Unit of Endocrinology, PhD School of Clinical and Experimental Medicine, University of Modena and Reggio Emilia, 42121 Reggio Emilia, Italy; 3 Fertility Clinic at Skive Regional Hospital, Faculty of Health, Aarhus University, 8000 Aarhus, Denmark; peter.humaidan@midt.rm.dk; 4 Department of Biological Sciences, Wichita State University, Wichita, KS 67260, USA; george.bousfield@wichita.edu; 5 Laboratory of Endometrium, Endometriosis & Reproductive Medicine, Department of Development and Regeneration, KU Leuven, 3000 Leuven, Belgium; 6 Department of Obstetrics, Gynecology and Reproductive Sciences, Yale University Medical School, New Haven, CT 06510, USA; 7 Red de Apoyo a la Investigación, Universidad Nacional Autónoma de México (UNAM)-Instituto Nacional de Ciencias Médicas y Nutrición SZ, Mexico City 14080, Mexico; aulloaa@unam.mx

**Keywords:** follicle-stimulating hormone, glycoform, gonadotropin, heterogeneity, potency, reproduction, sialylation

## Abstract

Follicle-stimulating hormone (FSH), together with luteinizing hormone (LH) and human chorionic gonadotropin (hCG), plays a fundamental role in human reproduction. The discovery of FSH and other gonadotropins was a defining moment in our understanding of reproduction and led to the development of many treatments for infertility. In this regard, exogenous FSH has been used to treat infertility in women for decades. Today, several recombinant and highly purified urinary forms of FSH are used in medically assisted reproduction (MAR). However, differences in the macro- and micro-heterogeneity of FSH result in a variety of FSH glycoforms, with glycoform composition determining the bioactivity (or potency), pharmacokinetic/pharmacodynamic (PK/PD) profiles, and clinical efficacy of the different forms of FSH. This review illustrates how the structural heterogeneity of FSH glycoforms affects the biological activity of human FSH products, and why potency does not predict effects in humans in terms of PK, PD, and clinical response.

## 1. Introduction

Follicle-stimulating hormone (FSH) is a glycoprotein hormone synthesized and secreted by the anterior pituitary gland under the pulsatile stimulus of the gonadotropin-releasing hormone (GnRH) peptide [1]. This gonadotropin, together with luteinizing hormone (LH) and human chorionic gonadotropin (hCG), plays a central role in mammalian reproduction. The first observations that reproductive function is regulated by the pituitary gland arose from the in vivo studies of Crowe et al. in 1910 [2,3]. These findings were confirmed 2 years later by Bernard Aschner, who also postulated that pituitary (gonadotropic) extracts might have practical applications [3,4]. This was followed by the discovery of the “gonadotropic principle” by Smith and Engle and Bernhard Zondek, independently of each other, who established that ovarian function is regulated by the pituitary gland [3,5,6]. In 1930, Smith went on to demonstrate that removal of the pituitary gland from adult rodent models without injury to the brain resulted in profound atrophy of genital organs, rapid regression of sexual characteristics, and total loss of reproductive function in both males and females [3,7]. Just prior to this in 1929, Zondek proposed the idea that the pituitary gland secretes two hormones, “Prolan A” and “Prolan B”, that stimulate the gonads; in 1930, he demonstrated that the blood and urine of postmenopausal women contained gonadotropins [3,8]. Zondek postulated that Prolan A stimulated follicular growth, Prolan A together with Prolan B stimulated the secretion of “folliculin”, and Prolan B induced ovulation, the formation of the corpus luteum, and the secretion of lutein and folliculin. Zondek’s hypotheses were confirmed with the extraction of two different hormones from the pituitary gland by Fevold et al., one of which acted as a follicle-stimulating factor and the other as a luteinizing factor [3,9]. Prolan A and Prolan B, therefore, became known as FSH and LH, respectively. Prior to this, in 1927, Ascheim and Zondek demonstrated that the blood and urine of pregnant women contained a gonad-stimulating substance (known today as hCG) that induced both follicular maturation and ovarian stromal luteinization and hemorrhage when injected into immature female mice; this became known as the Ascheim–Zondek pregnancy test [3,10]. Gonadotropins were among the first “biologically active ingredients” to be isolated and purified from biological fluids. Their discovery was a turning point in our understanding of reproduction and led to the development of fertility treatments for infertile patients [3].

Exogenous FSH has been used to treat infertility in women since the 1960s. The first preparations available for clinical use were extracted from the pituitary glands of animals and from the urine of postmenopausal women (i.e., “human menopausal gonadotropin” [hMG]). However, since these extracts were a mixture of gonadotropins, they were associated with safety concerns. Pituitary extracts induced an immune response and, hence, the production of antibodies that blocked their gonadotropic effects, while hMG had a low purity and contained many non-gonadotropin contaminants and oxides [11,12]. Technological advances led to the development of highly purified human urinary gonadotropins (HP-hMG and HP-FSH) suitable for therapeutic use. Thanks to the advent of recombinant DNA technology, the first recombinant human FSH (r-hFSH; follitropin alfa) preparation was produced by inserting the genes encoding the alpha and beta subunits of FSH into expression vectors that were transfected into Chinese Hamster Ovary (CHO) cell lines [12]. The main advantages of follitropin alfa are its high purity and batch-to-batch consistency [13]. The development of follitropin alfa in the 1990s remains the most significant breakthrough in drug development for assisted reproduction technology (ART), since it paved the way for the development of other recombinant proteins, including LH, hCG, and other r-hFSH products (i.e., follitropin beta, follitropin delta, follitropin epsilon, and follitropin alfa biosimilars, as well as the chimeric protein corifollitropin alfa).

Recombinant and highly purified urinary human FSH (HP-u-hFSH) products are used to stimulate follicular development. Nevertheless, some differences in terms of clinical outcomes have been detected when comparing the different products [14,15,16,17,18,19,20]. Such differences may depend on the structure of the glycans attached to the FSH protein core. Indeed, FSH is a complex glycoprotein that is expressed and secreted in different glycoforms, characterized by structural differences in the glycosylation resulting from post-translational modifications. It is known that glycan structure determines the biological activity, receptor binding, and PK properties (half-life and clearance) of the FSH molecule [21,22,23]. A full explanation of the terms used throughout this manuscript can be found in Appendix A.

The aim of this review was to illustrate how the structural heterogeneity of FSH glycoforms impacts the biological activity of FSH-based products, and to clarify why different products may exert particular effects in humans in terms of PK/PD and clinical response.

## 2. Physiological Relevance and Functional Significance of FSH Heterogeneity

**Figure 1:**
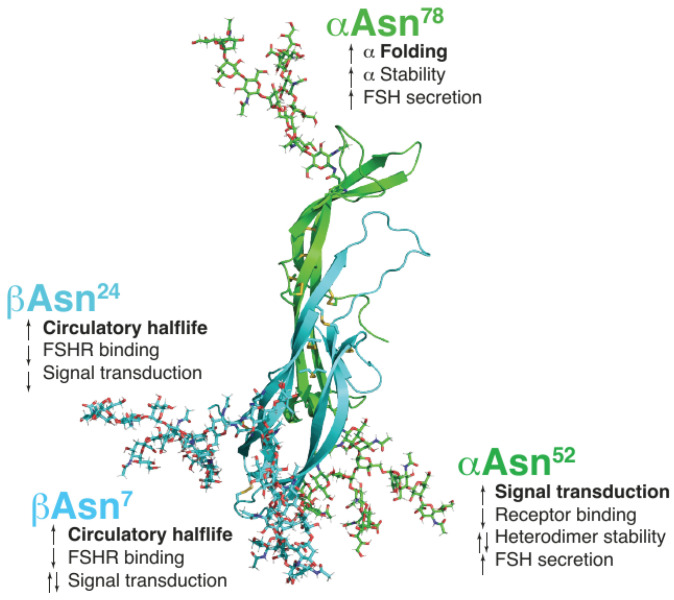
FSH consists of two subunits, the alpha subunit, common to all glycoprotein hormones, and the hormone-specific beta subunit. There are four glycan asparagine-linked glycosylation sites, two on the α subunit (green) and two on the β subunit (blue). Four FSH glycoforms that differ in the occupancy of the glycosylation sites were identified by FSHβ-specific Western blot analysis. FSHβ possessing both N-glycans and migrating as a 24 KDa band is designated “FSH^24^”. Partially glycosylated variants lack one or the other glycan on the β-subunit: FSH^18^ and FSH^21^ provide the 18 and 21 kDa bands, which represent the absence of either the Asn^7^ or Asn^24^ glycan, respectively, whereas FSH^15^ corresponds to the non-glycosylated FSHβ form that migrates as a 15 kDa band [24]. Adapted from Bousfield GR, et al. In vivo and in vitro impact of carbohydrate variation on human follicle-stimulating hormone function. *Front. Endocrinol. (Lausanne)* **2018**, *9*, 216; Copyright © 2018 Bousfield, May, Davis, Dias, and Kumar (Creative Commons Attribution License [CC BY]).

Like all glycoprotein hormones, FSH consists of two distinct noncovalently linked subunits, namely, α and β (Figure 1) [24]. While the α-subunit is common to all glycoprotein hormones (FSH, LH, hCG, and thyroid-stimulating hormone TSH), the β-subunit is distinct for each hormone and determines receptor specificity and biological and immunological properties [25]. Each subunit has two N-linked glycosylation sites; the α-subunit is glycosylated at both sites [26], whereas the sites on the β-subunit may or may not be occupied by glycans [21,22,24]. The attachment of N-linked and O-linked glycans to proteins and the extent of glycosylation determine the three-dimensional configuration of glycoproteins, thereby resulting in a variety of glycoforms that differ in structural stability and function. Glycosylation is critical for the action of glycoproteins, as well as to determine their PK and PD [21].

Absence of one or more oligosaccharide chains in a hormone results in macro-heterogeneity, which, in the case of FSH, is due to variations in glycan occupancy of the β-subunit (the α-subunit is always glycosylated at both Asn^52^ and Asn^78^). According to the glycan occupancy of the β-subunit, four FSH glycoforms have been identified: FSH^24^ is tetra-glycosylated and possesses all four N-glycans; FSH^21^ and FSH^18^ are tri-glycosylated forms that lack the βAsn^24^ glycan or βAsn^7^ glycan, respectively; the di-glycosylated FSH^15^ form lacks both glycans on the β-subunit [21] (Figure 1). Notably, FSH^15^ is not secreted by the pituitary [24]. The presence or absence of FSH β-subunit glycans modifies FSH properties (Table 1) [21,22,24]. The presence of βAsn^24^ increases circulatory half-life and reduces FSH binding to the FSH receptor (FSHR) and FSHR-mediated signal transduction compared with the FSH glycoform lacking glycosylation at βAsn^24^; βAsn^7^ increases circulatory half-life and reduces FSHR binding compared with the FSH glycoform lacking glycosylation at βAsn^7^, whereas the effect on signal transduction has yet to be established [24]. Consequently, FSH^21^ and FSH^18^ have shorter half-lives and higher receptor binding activity than the fully glycosylated FSH^24^. The types of FSH glycoforms naturally secreted vary across the menstrual cycle and other physiological states [27,28,29].

**Figure 2:**
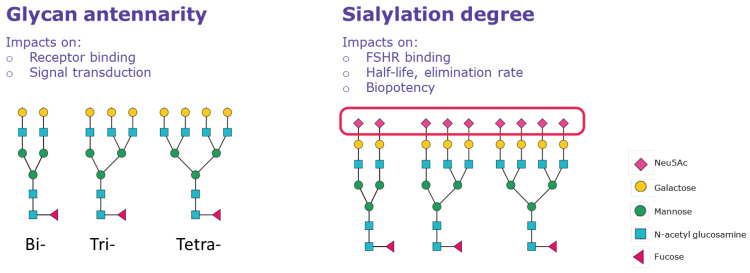
Micro-heterogeneity of FSH molecules and their impact on the PK/PD of hFSH products. All N-glycans are assembled on the endoplasmic reticulum (ER) membrane as a triantennary precursor Glc3Man9GlcNAc2 [37]. The glycan is added en bloc to FSH subunits as each sequon enters the ER lumen. The three glucose (Glc) residues and a single mannose (Man) residue are removed in the ER, and all but three Man residues and both N-acetyl glucosamine (GlcNAc) residues are removed in the Golgi, thereby leaving a two-antenna core. The antennae are initiated with GlcNAc and extended by galactose, the latter of which can be capped with sialic acid (Neu5Ac).

**Figure 3:**
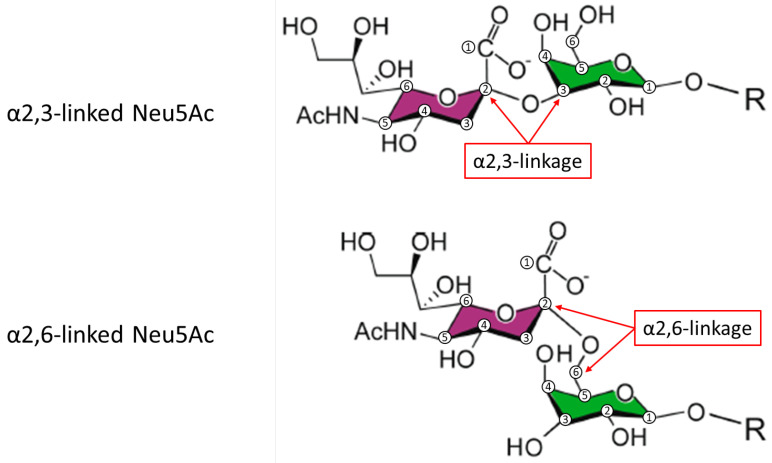
Sialic acid (N-acetyl neuraminic acid [Neu5Ac]) in α-2,3-linkage to galactose (**top panel**) and α-2,6-linkage to galactose (**bottom panel**). The areas shaded purple are the Neu5AC moieties, and the areas shaded green are the galactose moieties. Numbers in circles denote the carbon atom numbers [38]. Adapted from Byrd-Leotis L, Cummings RD, Steinhauer DA. The interplay between the host receptor and influenza virus hemagglutinin and neuraminidase. *Int. J. Mol. Sci.* **2017**, *18*, 1541; Creative Commons Attribution 4.0 International License, © 2017 by the authors.

Micro-heterogeneity is defined as the occurrence of variations in the structure of glycans attached on both the α and β subunits and represents a further level of complexity of FSH glycoforms that potentially affects their action (Figure 2 and Table 1). It depends mainly (but not exclusively) on the number of glycan branches (antennarity), on the carbohydrate residues, and on the presence or absence of galactose and sialic acid (sialylation) in the oligosaccharides attached to the protein core [30]. Antennarity impacts the binding of FSH to its receptor: bulky and extended glycans may result in a delayed receptor response while relatively smaller and more compact FSH glycans, e.g., the biantennary at αAsn^52^, have more rapid FSHR binding [21]. Antennarity indirectly influences the charge of FSH, due to the presence of sialic acid (N-acetyl neuraminic acid or Neu5Ac) moieties that cap the terminal end of N-glycans, rendering the FSH molecule more acidic. The greater the number of antennae, the greater the probability of having complete terminal sialylation (i.e., with both galactose and sialic acid attached). Neu5Ac can be added almost exclusively if a galactose residue is present in the carbohydrate chain of the terminal branch, and it may be attached to galactose via α2,3 or α2,6 linkage (Figure 3). Pituitary, serum, and urinary derived FSH contains both α2,3 and, to a lesser degree, α2,6 linkages. However, follitropin alfa expressed in CHO cells contains sialic acid linked only through α2,3 linkages [31,32,33]. Consequently, follitropin alfa expressed using the CHO system will differ from endogenous FSH in the type of terminal sialic acid linkages. In contrast, as follitropin delta is expressed in a retinal human cell line (PER.C6), the glycan profile resembles that of urinary FSH, containing a higher proportion of tri- and tetra-sialylated glycans with both α2,3- and α2,6-linked sialic acid, compared with follitropin alfa produced by CHO cells, which does not contain the α2,6 linkage [34]. Although sequential addition of terminal N-acetylgalactosamine (GalNAc) and sulfate yields sulfated oligosaccharides [35], sialylated GalNAc residues instead of sialylated Gal have been detected in at least one antenna in complex biantennary, triantennary, and tetraantennary human FSH glycans [21], as also observed in bovine FSH [36]. Thus, the level of acidity is mainly determined by the presence or absence of sialic acid, resulting in different sialylated glycans, such as neutral and mono-, di-, tri-, and tetra-sialylated glycoforms.

Sialylation is the major factor influencing FSH in vivo clearance rate. More acidic/sialylated glycoforms, as determined by charge-based procedures, exhibit slower plasma elimination rates than less acidic forms due to reduced renal clearance [39]. In addition, glycoforms with α2,6-linked sialic acid result in slower elimination rates versus glycoforms with α2,3-linked sialic acid; the slower elimination rate may depend on the clearance mechanism of these different forms. α2,3-linked sialic acid is metabolized mainly by the kidneys, whereas α2,6-linked sialic acid is metabolized mainly by the asialoglycoprotein receptor (ASGPR) in the liver. However, expression of hepatic ASGPR is lower in humans than in mice or rats, suggesting less dependence on this clearance mechanism in humans [40], resulting in a lower serum clearance rate in humans of glycoforms with α2,6-linked sialic acid than the clearance rates observed in rodent models [19]. Consequently, if the same bioactivity and pharmacokinetic behavior of hFSH products with and without α2,6-linked sialic acid are demonstrated using the Steelman–Pohley in vivo rat bioassay, this might not translate into the same bioactivity demonstrated in humans [19].

FSH charge also affects receptor affinity: less acidic/sialylated glycoforms exhibit higher FSHR binding than more acidic/sialylated forms [22,30,41]. In summary, the receptor binding and PK properties (half-life and clearance) of FSH depend on both macro- and micro-heterogeneity (i.e., glycosylation, sialyation, and sulfation). Consequently, the particular glycoform composition of hFSH products may also have clinical implications.

## 3. FSH Heterogeneity during Pubertal Development and during Ovarian Cycles

The variability of pituitary FSH glycoforms during the menstrual cycle and with aging suggests that glycoform composition plays a functional physiological role. Glycoform variations are regulated by hormonal feedback from the ovaries. The increase in estradiol levels in the ovaries that occurs during the follicular phase and ends just before ovulation (between day 5 until the estradiol peak at day 14) stimulates the secretion of the less acidic and less glycosylated FSH^18^ and FSH^21^ glycoforms that have a shorter half-life and greater in vitro biological activity than the fully glycosylated FSH^24^ form. The acidic glycoforms peak at the mid-follicular phase (days 5–9 of the menstrual cycle) [42]. Such forms have a lower receptor affinity and a prolonged in vivo half-life due to reduced renal clearance [43,44]. Furthermore, a progressive reduction in the hypoglycosylated FSH^18^ and FSH^21^ glycoforms is found in aging women [24], thereby resulting in an increased prevalence of FSH^24^ glycoforms in menopausal women [45,46].

## 4. Biological Activity and Potency of hFSH Products

The measurement of biological activity, namely, potency (expressed in international units [IU] or micrograms [μg]), indicates the specific ability of a product to achieve a predefined biological effect [47]. When the measure of biological activity is obtained using a bioassay, it is referred to as biopotency expressed in IU. Potency is a relative measure that depends on the assay used to determine it. The Steelman–Pohley in vivo bioassay is the standard procedure employed to measure FSH product biopotency [48]. It is based on the linear relationship between increasing doses of FSH (daily dose in IU for 3 days) and the increase in ovarian weight of immature rats versus a reference standard [48]. Originally, the reference standard was hMG; this was replaced by follitropin alfa [49]. The accepted coefficient of variation for each determination is between 10% and 20%, meaning that, considering the maximum variation (20%), by injecting 100 IU of the FSH product, the actual bioactivity found in the assay can be any value between 80 and 120 IU [50]. Recently, however, an in vitro bioassay demonstrated similar ability to the Steelman–Pohley in vivo bioassay to detect chemical/physical differences in r-hFSH variants that strongly impact biopotency [51,52]. Notably, the in vitro bioassay was recently approved by the European Medicines Agency (EMA) to replace the in vivo bioassay after obtaining a positive opinion from the Committee for Medicinal Products for Human use (CHMP) on 27 October 2022 for originator follitropin alfa (GONAL-f^®^) and follitropin alfa/lutropin alfa (Pergoveris^®^) [53]. Therapeutic proteins with high purity and batch-to-batch consistency can be quantified by physicochemical methods in order to determine protein content (“mass”) [54]. In some cases, potency is expressed in mass [54]. Although biopotency in IU is the standard to determine the FSH product dose for clinical use, r-hFSH products can also be quantified by the mass of the purified product (Table 2).

### 4.1. Measurement of Potency of hFSH Products

Even though it is possible to quantify the total protein content (“mass”) in urinary gonadotropins, it is a challenge to assess only the FSH protein content quota; therefore, the potency of urine derived FSH molecules is usually expressed in IU determined using the Steelman–Pohley bioassay [48]. Urinary gonadotropins comprise a mixture of gonadotropins (FSH, pituitary and urinary hCG, and LH), and they also contain up to 20% of non-gonadotropin proteins, as well as 40% of oxidized FSH forms [11,13,55]. A total of 23 serum proteins of non-gonadotropin origin were identified in hMG, the level of which differs among batches [11].

By contrast, follitropin alfa is highly purified and has a high batch-to-batch consistency. The potency of follitropin alfa can be determined using either the Steelman–Pohley in vivo bioassay or a recently developed in vitro bioassay [51,52]. The FSH content can be quantified using chromotagraphic techniques, where analysis of the protein mass considers the entire protein, including the oligosaccharide side chains [50]. A fixed conversion factor has been established to determine the potency using the protein content; therefore, measurement of the FSH protein content is sufficient to estimate the bioactivity in the medicinal product), where analysis of the protein mass considers the entire FSH glycoprotein, including the oligosaccharide side chains. Follitropin alfa-containing medicinal products are, however, still labeled and dosed in terms of IU, as clinicians still use the biological activity to individualize the dose that will provide the optimal clinical response for each patient. As glycosylation affects both the glycoprotein mass and the biological activity of recombinant FSH preparations, any differences in glycosylation between individual batches are accounted for by the manufacturer when calculating the conversion factor between mass and biological activity. The conversion factor describes the amount of protein that is equivalent to 1 IU (µg/IU) and is established based on the manufacturing history (i.e., it averages out the variability in glycosylation between individual batches). As discussed in the next section, follitropin delta is labeled only by mass, since the biological activity in IU measured by the Steelman–Pohley bioassay in rats does not accurately reflect the PK/PD in humans [19].

A further level of complexity is that the same dose (in IU) of different hFSH products does not translate into similar PK/PD in humans. Indeed, biopotency determined in the rat in vivo Steelman–Pohley bioassay is a comparative rather than an absolute expression of drug activity that, in the case of hFSH products, is influenced by macro- and micro-heterogeneity. This, in turn, is a consequence of hFSH source, production, and culture conditions [22], resulting in differences in renal clearance due to differences in hepatic metabolism between animal models and humans [40]. As observed in clinical studies in women, a higher biopotency of hFSH according to the rat in vivo Steelman–Pohley bioassay does not necessarily imply a stronger or better clinical response to exogenous FSH in humans [19], as discussed in the section on physiological relevance and functional significance of FSH heterogeneity. Instead, the actual efficacy of hFSH in humans results from the complex interaction of several factors, including hFSH plasma half-life and the interaction of hFSH with its receptor [56].

### 4.2. hFSH Products Used in Medically Assisted Reproduction

#### 4.2.1. Urinary Gonadotropins

Urinary gonadotropins (u-hFSH and hMG) mainly consist of fully glycosylated (hFSH^24^) glycoforms [22], with a predominance of highly sialylated and highly branched glycans possessing both α2,3- and α2,6-linked sialic acid [22]. Therefore, urinary derived FSH has a longer half-life and reduced FSHR binding than drug substances from other origins that contain less branched glycoforms [22,45].

#### 4.2.2. Follitropin Alfa Originator (GONAL-f^®^)

Follitropin alfa originator, produced by recombinant DNA technology in a CHO cell line, contains ~5% neutral FSH glycoforms, 25% mono-sialylated FSH glycoforms, 50% di-sialylated FSH glycoforms, 15% tri-sialylated FSH glycoforms, and <5% tetra-sialylated FSH glycoforms [50]. Follitropin alfa contains only α2,3-linked sialic acids [22]. It has a high batch-to-batch consistency in terms of glycosylation profile and glycan species distribution [13,21]. Despite having a comparable PK/PD profile, follitropin alfa has a lower plasma half-life and higher receptor binding activity for the FSHR than urinary gonadotropins. This could explain why administering the same starting dose in IU of follitropin alfa and urinary gonadotropins results in a higher level of follicle growth and a higher number of oocytes after ovarian stimulation with follitropin alfa [14,57,58,59,60,61].

#### 4.2.3. Follitropin Alfa Biosimilars

Follitropin alfa biosimilars have recently been approved for clinical use. Follitropin alfa biosimilars are also produced by recombinant DNA technology in a CHO cell line. Follitropin alfa biosimilars contain only α2,3-linked sialic acids and differ from follitropin alfa originator owing to the structural complexity of glycoprotein molecules due to post-translational modifications [18,23,62,63]. Glycosylation analysis of the follitropin alfa biosimilar Ovaleap^®^ shows a slight shift in sialic acid content and an increase in nonhuman sialic acid variants containing N-glucolneuramic acid (Neu5Gc) [63], but lower total sialic acid content, compared with the originator. These differences might potentially lead to differences in binding to the FSHR and in circulating half-life. The follitropin alfa biosimilar Bemfola^®^ has bulkier glycan structures and greater sialylation than the originator [18]. Moreover, its glycan profile at Asn^52^, which activates FSHR signaling and influences heterodimer stability, has a lower proportion of biantennary structures and a higher proportion of tri- and tetra-antennary structures than the originator [18]. Various differences in N-glycosylation occupancy, antennarity, and sialylation, as well as minor differences in oxidation levels, were also detected between originator and other biosimilar preparations (namely, Primapur^®^, Jin Sai Heng^®^, Corneumon^®^, Folisurge^®^, and Follitrope^®^) [23].

#### 4.2.4. Follitropin Beta (Puregon^®^)

Follitropin beta is also produced in CHO cells. Its glycosylation profile is very similar to that of follitropin alfa, and it contains only α2,3–linked sialic acids [22]. The specific activity declared by the manufacturer is 10,000 IU/mg [64], whereas the specific activity for follitropin alfa is 13,636 IU/mg [18]. As a result of the post-translational glycosylation process and purification procedures, the two preparations are not identical in terms of sialic acid residues and isoelectric point; follitropin alfa is slightly more acidic than follitropin beta [13,65]. As reported in Table 2, the follitropin beta manufacturing process specifies that this is filled by IU [64]. The available published evidence does not provide a conversion factor between mass and biological activity for follitropin beta, as described in this paper for follitropin alfa (Section 4.1).

#### 4.2.5. Follitropin Delta (Rekovelle^®^)

Follitropin delta is produced by recombinant DNA technology in human PER.C6 cells. It contains both α2,3- and α2,6-linked sialic acids. The higher sialic acid content of follitropin delta and the presence of both α2,3- and α2,6-linked sialic acids results in increased charge and size of follitropin delta compared with follitropin alfa, as well as lower renal clearance and slower clearance from serum due to hepatic metabolism of α2,6-linked sialic acids. As reported in the follitropin delta patent [34], the parent clone originally contained only α2,6-linked sialic acid; however, because it failed to reach the biopotency of follitropin alfa, it was re-engineered by adding α2,3 linkages. The resulting molecule with both α2,3- and α2,6-linked sialic acids has a biopotency similar to that of follitropin alfa, as assessed in the Steelman–Pohley rat in vivo bioassay [16]. Nevertheless, phase 1 studies on the PK/PD of follitropin delta showed that follitropin delta was not comparable to follitropin alfa in women when equivalent doses in IU of the two products were tested [19]. The different PK/PD behavior could be attributed to the rat animal model used to assess biopotency in IU. As previously explained, in humans, the α2,3-linked sialic acid is metabolized mainly by the kidneys, whereas α2,6-linked sialic acid is metabolized mainly by the ASGPR in the liver, which has a lower expression in humans than in rodents, thereby resulting in a lower serum clearance rate of follitropin delta in humans when compared with rats or mice [19,40]. Thus, dosing of follitropin delta in IU results in a discrepancy between the expected bioactivity (based on the rat in vivo bioassay) and the actual clinically observed ovarian response in humans, consequently leading to a potential risk of developing OHSS in women [66]. Based on these findings, follitropin delta doses are expressed by protein content (μg) and not by bioactivity (IU) [67], and the starting dose is determined using an algorithm that considers the patient’s anti-Müllerian hormone (AMH) serum level and body mass index (BMI) based on a predictive model proposed by the manufacturer [68].

## 5. Effects of Glycoform Composition on the PK/PD of FSH Preparations

As discussed above, glycan structure is a determinant of the PK/PD of FSH preparations in terms of circulatory half-life, in vivo bioactivity, and receptor binding to the FSHR [30].

### Glycoform Composition Is Related to the Clinical Effect

Follitropin alfa and follitropin beta are characterized by the presence of twofold fewer acidic glycans than urinary FSH. In humans, the higher content of sialic acid confers a longer in vivo half-life to urinary gonadotropins compared with follitropin alfa and beta. Despite differences in terminal glycosylation and the longer half-life, the PK profiles of urinary gonadotropins and of recombinant follitropin alfa products produced by CHO cells are still within the acceptance criteria and are considered similar in terms of PK behavior [64,69,70,71]. Compared with a single injected dose of urinary FSH, a single injected dose of a recombinant follitropin alfa preparation resulted in lower immunoreactive serum FSH levels, but higher circulating bioactive FSH concentrations [72]. This explains why, as we discuss in the next section, ovarian stimulation with recombinant follitropin alfa has been reported to require lower total and daily doses and a shorter treatment period prior to triggering follicular maturation than urinary gonadotropins [14,58]. This observation from clinical practice clearly illustrates that longer half-life does not translate into higher clinical efficacy in humans [30,73].

**Figure 4:**
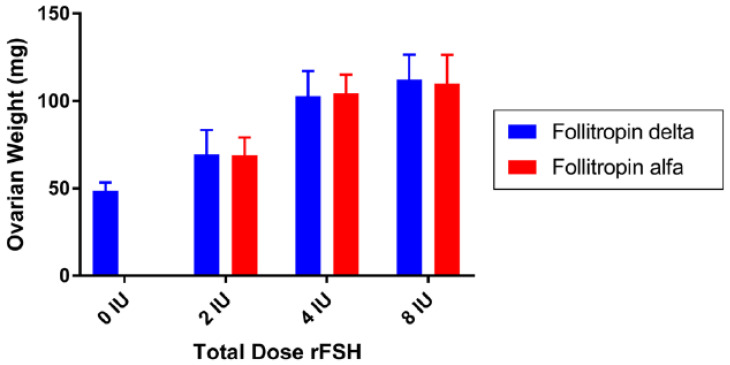
The bioactivity of follitropin delta (blue bars) and follitropin alfa (red bars) was compared in vivo in the rat in the Steelman–Pohley bioassay, measuring the increase in ovarian weight with the administration of the indicated total dose of rFSH proteins. Bars are the mean of 14 animals in each dose group, and error bars are the standard deviation [16]. Taken from Koechling W, et al. Comparative pharmacology of a new recombinant FSH expressed by a human cell line. *Endocr. Connect* **2017**, *6*, 297–305; Creative Commons Attribution 4.0 International License.

The glycosylation profiles of follitropin delta and follitropin alfa differ substantially in terms of sialylation, fucosylation, and antennarity across all N-glycosylation sites [74]. Follitropin delta has a higher proportion of tri- and tetra-sialylated glycans than follitropin alfa [74]. In addition, follitropin delta has both α-2,3- and α-2,6-linked sialic acid, while follitropin alfa has only α-2,3-linked sialic acid [74]. The dose–response curves of follitropin alfa and delta in vitro using fresh, luteinized granulosa cells from IVF patients and in the human embryonic kidney 293 (HEK-293) cell line expressing the FSH receptor were comparable [16]. Both follitropins also exhibited very similar pharmacodynamic behavior in the rat when compared using the Steelman–Pohley bioassay (Figure 4) [16]. However, when testing these follitropins in ASGPR knockout mice versus wildtype, the elimination of ASGPR reduced clearance of follitropin delta but not of follitropin alfa in the knockout mice, suggesting that follitropin delta but not follitropin alfa is eliminated via the liver ASGPR system [16]. In humans, the clearance of repeated doses (225 IU subcutaneously) of follitropin delta was lower than that of follitropin alfa (0.58 vs. 0.99 L/h, respectively), the area under the curve and the maximum serum concentration were higher (1.7-fold and 1.6-fold, respectively), and the elimination half-lives were approximately 30 h for follitropin delta and 24 h for follitropin alfa [19]. PD also differed between the two preparations in terms of number of follicles and serum concentrations of inhibin B and estradiol, which were higher with follitropin delta than with follitropin alfa at the same daily dose (225 IU) [19]. As discussed, the different PK profiles of follitropin delta versus follitropin alfa are hypothesized to depend on the hepatic ASGPR metabolism of α2,6-linked sialic acid in follitropin delta [16]. However, this hypothesis should be taken with caution, since although urinary FSH contains both α2,3- and α2,6-linked sialic acids, the PK profile of urinary FSH is comparable to that of follitropin alfa, as already discussed [16]. It is, therefore, conceivable that the PK/PD profile of follitropin delta, which is not comparable to other hFSH preparations, depends not only on α2,3-linked and α2,6-linked sialic acid content, but also on other differences in glycosylation [74]. Furthermore, this hypothesis does not account for the fact that single injections of identical units of biological activity of follitropin delta result in a higher ovarian response in humans compared with follitropin alfa [19]. This probably explains why dose adjustment during ovarian stimulation for ART is not allowed within the label according to the Rekovelle SmPC [67], whereas it is permitted for other r-hFSH products, such as follitropin alfa [70] and follitropin beta [64].

Despite what has been previously hypothesized [34], the PK/PD differences between follitropin alfa and follitropin delta may not be fully due to the presence of the α2,6-linked N-acetylneuraminic linkage. In fact, although follitropin delta is produced in a human cell line, compared with the beta subunit of pituitary FSH, the beta subunit of follitropin delta has been reported to have a different α2,6 N-acetylneuraminic linkage distribution and a shift toward higher tri- and tetra-sialylated glycans [74]. In addition, as observed in PK/PD studies of follitropin alfa versus urinary gonadotropins, the presence of α2,6-linked N-acetylneuraminic in the urinary gonadotropin did not result in a significantly different PK between the two products [69,71]. Accordingly, reported differences in the glycosylation profiles, antennarity, and sialylation and fucosylation levels among pituitary-derived FSH in urine, follitropin alfa and follitropin delta (with differences observed at N-glycosylation sites on both the alpha subunit [directly involved in receptor interaction and activation] and the beta subunit [important for circulatory half-life]) may better explain the PK/PD differences [74]. Interestingly, r-hCG from PERC.6 cells (CG beta) also exhibits different PK/PD profiles in humans compared with r-hCG from CHO cell lines (CG alfa) and u-hCG. In particular, there is a difference in PK between the two r-hCG preparations, causing increased exposure and greater PD response for CG beta when compared to CG alfa [75].

## 6. Effect of the Glycoform Composition of hFSH Preparations on Clinical Response

Glycoform composition determines the bioactivity, the PK/PD in humans, and, ultimately, the clinical efficacy of hFSH products. Randomized clinical trials (RCTs) comparing different hFSH products consistently revealed discordant results for various outcomes [14,58,76]. Although there is no difference in terms of clinical efficacy among hFSH products [77], different clinical outcomes may be relevant for physicians seeking to personalize treatment. Clinical response must be evaluated in a selected patient population using appropriate outcome parameters (e.g., number of oocytes, pregnancy rate, live birth rate, cumulative pregnancy rate, and cumulative live birth rate). Moreover, outcome measures also depend on treatment goals. For example, in clinical development, according to the European Medicines Agency, oocyte number is the most appropriate outcome measure to compare efficacy in terms of follicular development for r-hFSH and its biosimilars [78]. In contrast, the ESHRE Guideline on Ovarian Stimulation for IVF/ICSI considers cumulative live birth or live birth as the most appropriate outcome to measure efficacy [77]. The latter outcomes pose practical challenges for clinicians and researchers due to the length of follow-up and the variables that may eventually affect the clinical results, such as number of embryos transferred and maternal age.

### 6.1. Efficacy Outcomes of hFSH Products

#### 6.1.1. Urinary Gonadotropins versus Follitropin Alfa

RCTs comparing the same starting dose of urinary gonadotropins versus follitropin alfa (225 IU or 150 IU) consistently found that treatment with urinary gonadotropins resulted in fewer oocytes with a higher total dose and longer treatment duration than follitropin alfa [14,58,70,76]. When considering the number of oocytes retrieved, 1 IU of follitropin alfa results in a higher clinical response than 1 IU of urinary gonadotropins [14,58,70]. Therefore, the longer half-life of urinary gonadotropins versus follitropin alfa does not translate to a greater clinical response, but instead results in a lower number of oocytes and embryos. This may be explained by the relatively lower affinity of urinary gonadotropins for the FSHR compared with follitropin alfa, due to the higher number of fully glycosylated glycoforms [45].

The gonadotropin starting dose is an important determinant of the number of oocytes retrieved [79], and RCT evidence consistently reports that, at the same starting doses, originator follitropin alfa systematically yields more oocytes and/or embryos than urinary gonadotropins [14,57,58,59,60,61]. The added value of more oocyte/embryo numbers retrieved with r-hFSH compared with urinaries may be the higher cumulative live birth rates, regardless of the starting dose/total dose, as reported in studies based on real-world data [80,81].

#### 6.1.2. Follitropin Alfa Originator versus Follitropin Alfa Biosimilars

When administered at the same dose, follitropin alfa biosimilars resulted in no difference in the number of oocytes compared with follitropin alfa originator. However, a meta-analysis of RCTs on efficacy and safety found a lower probability of live birth and pregnancy (ongoing and clinical) with similar doses in IU for follitropin alfa biosimilars versus follitropin alfa originator [15]. Furthermore, irrespective of the starting dose, real-world data on 245,534 stimulations in the French National Health Database showed a 19% lower live birth rate and a 14% lower cumulative live birth rate with follitropin alfa biosimilars compared with originator follitropin alfa [81].

#### 6.1.3. Follitropin Alfa Originator versus Follitropin Delta

RCTs on follitropin delta versus follitropin alfa originator revealed no differences in terms of the number of oocytes retrieved, ongoing pregnancy, and live birth rates [67,68,70,82,83]. However, the study design of these RCTs is questionable and is not suitable to establish noninferiority between the two products because they compared different dosing protocols; a fixed 150 IU dose of follitropin alfa originator (regardless of patient characteristics) was compared with a personalized dose of follitropin delta in μg, established according to patient age, BMI, and AMH levels [84,85]. Furthermore, a fair comparison between follitropin delta and follitropin alfa originator requires individualized starting doses in both arms [84,85].

In addition, given the PK/PD response to follitropin delta, it is not possible to establish dose equivalence with other hFSH products that are dosed in IU. This is an important limitation for its clinical use in terms of reliability and predictability of outcomes, since the extent to which the differences in PK/PD will be reflected in clinical practice cannot be predicted. A post hoc analysis of ovarian response data from a phase 2 dose–response trial [86] and a phase 3 efficacy trial [68] attempted to establish the daily dose of follitropin delta (in µg; range 5–12 µg) that provides the same biological response as 150 IU/day follitropin alfa [66]. A linear relationship between log dose for follitropin delta and ovarian response was assumed. For the phase 3 data, the patients randomized to a 150 IU/day fixed dose of follitropin alfa were categorized in subgroups based on their AMH concentrations and body weight and matched to the dose of follitropin delta they would have received. The follitropin delta dose leading to a comparable ovarian response to 150 IU/day of follitropin alfa was determined as the intersection of the linear regression lines of follitropin delta and follitropin alfa groups.

**Figure 5:**
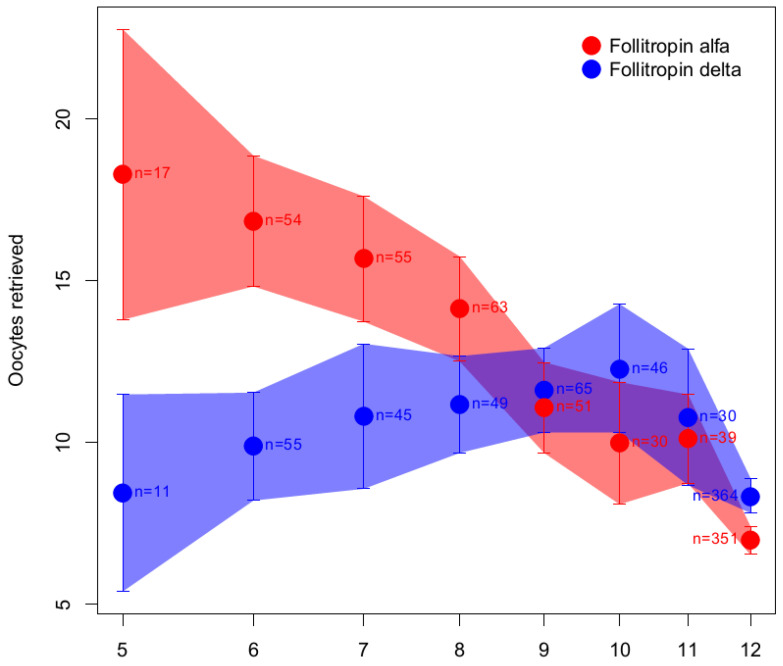
Number of oocytes retrieved for follitropin delta and 150 IU/day follitropin alfa in the phase 3 efficacy study (NCT01956110, [68]). Estimated means (dots) and 95% confidence intervals (95% CI) with number of patients for subgroups based on the dose of follitropin delta corresponding to the patients’ AMH concentration and body weight. The area between the upper and lower bounds of the 95% CIs is shaded to give an illustration of the shape of the data [66]. Reproduced from Arce JC, Larsson P, García-Velasco JA. Establishing the follitropin delta dose that provides a comparable ovarian response to 150 IU/day follitropin alfa. *Reprod. Biomed. Online* **2020**, *41*, 616–622. Copyright © 2020, with permission from Elsevier.

For the purposes of clear illustration, we replotted the follitropin delta dose subgroups showing the full data range based on the mean and 95% confidence interval values provided in the original article [66] (Figure 5). As shown in Figure 5, it is clear that the follitropin delta dosing area where the data (number of oocytes retrieved) overlap with the data obtained after daily dosing with 150 IU follitropin alfa (the area shaded purple in Figure 5) is wide and ranges from 8 to 12 µg of follitropin delta.

Figure 5 illustrates that any dose between 8 and 12 µg of follitropin delta could potentially be considered the dose estimated to have the same response as that proposed by Arce et al. (10 µg) [66]. Furthermore, it is apparent from Figure 5 that the relationship between the log dose of follitropin delta and the response is not linear, which raises doubts about the interpretability of the conclusion drawn by the authors using a linear approximation [66]. In conclusion, the approach to claim that the doses at the intersection of the linear function are comparable [66] is invalid as the model does not fit the data. Moreover, the question remains as to whether the number of oocytes retrieved is the most relevant endpoint to demonstrate the fixed correlation between IU assessed using the rat in vivo bioassay and µg of follitropin delta when PK/PD equivalence was not demonstrated when performed according to the same principle.

In conclusion, the findings of Arce et al. [66] cannot be generalized to IVF/ICSI patients since the dose comparison provided was based on noncomparable arms (individualized vs. fixed dose) and resulted in a range of “equivalent” doses, instead of a single equivalence dose. Such an approach could have consequences in terms of patient safety in clinical practice. This topic should be more accurately addressed in a well-designed, dedicated clinical study to compare ovarian response, pregnancy rates, and other clinically relevant parameters (e.g., endometrial thickening), and taking into consideration the PK/PD differences between the two molecules reported by Arce et al. [66] and discussed in this review. Most importantly, any comparison between follitropin alfa and follitropin delta would be scientifically sound and clinically relevant only if both regimens are individualized [85].

## 7. Conclusions

FSH is a complex molecule; differences in macro- and micro-heterogeneity result in a variety of FSH glycoforms that impact the biological effects at the target cell level. Moreover, heterogeneity of FSH glycoforms determine plasma half-life and clearance [21,22]. Thus, such variety is of clinical importance and may explain the discrepancies observed in PK/PD and in clinical outcomes using different hFSH products. The same dose in IU of urinary hFSH products results in fewer oocytes versus follitropin alfa and follitropin beta. A meta-analysis of RCTs using the same starting dose in both arms reported that the follitropin alfa originator results in higher live birth rates versus its biosimilars [15]. However, further studies are needed to confirm these findings. Regarding follitropin delta, despite various attempts, a reliable method to compare follitropin delta with other hFSH products has yet to be established. However, an equivalence cannot be made by comparing individualized starting doses of follitropin delta with fixed starting doses of follitropin alfa or other FSH molecules [84,85,87].

The differences in clinical effects observed, as well as the different modes of dose determination of the various hFSH products used for ovarian stimulation, are a challenge to clinicians when selecting the best treatment, the best starting dose, and the dose adjustment policy during ovarian stimulation for each patient. Currently, it is not possible to establish a dose equivalence in IU or mass that reflects clinical efficacy across all hFSH products and further studies are needed to establish equivalence among the different products. In this context, the appropriate selection and characterization of patients, the optimization of trial design, and the selection of appropriate endpoints are crucial.

In the future, it can be hypothesized that new products containing mixtures of hFSH^18/21^ with hFSH^24^ at different ratios may perform better than the currently available products, as they will mimic more closely the changes in the endogenous FSH stimulus in normal physiological conditions [27,29].

## Figures and Tables

**Table 1:** Structural and functional heterogeneity of FSH glycoforms.

Heterogeneity	Structural Feature	Glycoform Properties
Macro-heterogeneity	β-subunit glycan occupancy	**FSH^18,21^** ↑ FSHR affinity ↑ in vitro biological activity ↓ half-life ↑ plasma clearance **FSH^24^** ↓ FSHR affinity ↓ in vitro biological activity ↑ half-life ↓ plasma clearance
Micro-heterogeneity	Terminal branches antennarity	**High degree of antennarity** ↓ FSHR binding (delayed receptor response) **Low degree of antennarity** ↑ FSHR binding (rapid receptor response)
	Sialylation (acidity/sulfation)	**More acidic forms** ↓ FSHR binding ↑ half-life ↓ elimination rate **Less acidic forms** ↑ FSHR binding ↓ half-life ↑ elimination rate

FSH, follicle-stimulating hormone; FSHR, follicle-stimulating hormone receptor. ↑ Increased; ↓ Decreased.

**Table 2:** Labelling of FSH gonadotropin products.

	Follitropin Alfa	Follitropin Beta	Follitropin Delta	Urinary Products
**FSH content (quantity of protein by chromatographic methods)**	Dosed in μg	NA	Dosed in μg	NA
**Biopotency of specimen according to biological activity Steelman–Pohley bioassay**	Dosed in IU	Dosed in IU	NA	Dosed in IU
Specific bioactivity * expressed in IU/mg FSH	13,636 IU/mg	10,000 IU/mg	NA	NA
**Filling process**	Filled-by-mass (μg of FSH protein) and labelled in μg and IU	Filled by IU	Filled-by-mass (μg of FSH protein)	Filled by IU

* Specific bioactivity is the ratio of biopotency, measured using the Steelman–Pohley bioassay, and the protein content, measured with SE-HPLC. IU, international unit; μg, micrograms; NA, not applicable; SE-HPLC, size exclusion high-performance liquid chromatography.

## Data Availability

Any requests for data by qualified scientific and medical researchers for legitimate research purposes will be subject to Merck KGaA’s Data Sharing Policy. All requests should be submitted in writing to Merck KGaA’s data sharing portal (https://www.merckgroup.com/en/research/our-approach-to-research-and-development/healthcare/clinical-trials/commitment-responsible-data-sharing.html (accessed on 14 May 2023)). When Merck KGaA has a co-research, co-development, co-marketing, or co-promotion agreement, or when the product has been out-licensed, the responsibility for disclosure might be dependent on the agreement between parties. Under these circumstances, Merck KGaA will endeavor to gain agreement to share data in response to requests.

## Appendix A

**Action (or mode of action):** In medicine, this describes how a drug or other substances produce an effect in the body. For example, a drug’s mechanism of action could be how it affects a specific target in a cell, such as an enzyme, or a cell function, such as cell growth [88]. **Antennarity:** The number of branches in glycans (bi-, tri-, or tetra-antennary), influences acidity and can impact binding to the FSHR. Indeed, bulky and extended glycans may result in a delayed receptor response while relatively smaller and more compact FSH glycoforms (e.g., a biantennary glycan at αAsn-52) have more rapid FSHR binding [21]. The higher the number of antennae, the greater the probability of having complete glycan moieties (i.e., with galactose and sialic acid attached). In fact, sialic acid can be added only if a galactose (or less commonly GalNAc) residue is present in the carbohydrate branch. Therefore, the number of antennae indirectly influences acidity through sialylation. Alternatively, sequential addition of N-acetylgalactosamine (GalNAc) and sulfate produces sulfated oligosaccharides [35]. **Biological activity:** The specific ability of a product to achieve a defined biological effect. A valid biological assay to measure the biological activity should be provided by the manufacturer [54]. This depends on the assay used to measure it. **Biopotency/potency:** The quantitative measure of biological activity based on the attributes of the product (e.g., the nature and quantity of product-related substances, product-related impurities, and process-related impurities) that is linked to the relevant biological properties (e.g., biological activity). A valid biological assay (e.g., in vitro bioassay or in vivo animal bioassay or biochemical assays) to measure the biological activity (biopotency) should be provided by the manufacturer of the product [54,89]. When a valid bioassay is not suitable to measure the biological activity, we refer to potency in terms of quantity of protein (see *Quantity*). **Clinical response:** The effect of the product after its administration in humans, which should be established in clinical studies in a given population against specific predefined clinical endpoints [54]. **Efficacy:** For treatment with assisted reproductive technology [90], the critical efficacy outlines are cumulative live birth rate per started cycle and live birth rate per started cycle. Additional efficacy endpoints include cumulative ongoing pregnancy rate per started cycle, clinical pregnancy rate per started cycle, number of oocytes retrieved, and number of MII oocytes retrieved [77]. **Glycoproteins:** Proteins that contain glycans covalently attached to amino acid side chains of Asn, Ser, or Thr residues. This post-translational process occurs in the cell endoplasmic reticulum (ER) and Golgi apparatus, and is known as “glycosylation” [91]. **Glycans (also called “oligosaccharides”):** Carbohydrate-based polymers that are essential for the structure, energy storage, receptor binding affinity, and clearance of glycoproteins. **Glycosylation:** The addition of glycans (N-linked and O-linked glycans) to proteins. It is a highly regulated mechanism of secondary protein processing within cells, and it affects the three-dimensional configuration of proteins, their intracellular traffic and secretion, and their function and stability. Glycosylation is critical for the action of glycoproteins, as well as for their pharmacokinetics and pharmacodynamics (PK/PD) [21]. **Glycoforms:** Variants of a glycoprotein that have different glycans attached to the same glycosylation sites and/or possess glycan-specific glycosylation sites [21]. **International unit (IU):** The amount of a substance that exerts a given biological effect. For each substance there is international agreement on the biological effect that is expected for 1 IU [88] following the potency of a reference international standard (see also *Biopotency/Potency*). **International IU of FSH:** The activity contained in a stated amount of the International Standard of follitropin alfa [92]. The equivalence in IU of the International Standard is stated by the World Health Organization (WHO) for any new certified International Standard [93]. The first WHO International Standard defined 1 IU as the amount of FSH exerting an activity corresponding to 0.11388 mg of pure human urinary FSH (in vivo rat bioassay Steelman–Pohley) [94]. Each new standard is calibrated and validated according to a WHO Expert Committee of International Standard. The replacement IU is defined by the contents of the ampoule of the new standard. Every effort is made to maintain the continuity of the IU, but the replacement IU is not formally traceable to the first International Standard [93]. **Isoform:** A protein isoform, or “protein variant”, is a member of a set of highly similar proteins that originate from a single gene or gene family and are the result of genetic differences. An isoform may have differences in primary structure and/or post-translational modifications (e.g., glycosylation), or both. **Medically assisted reproduction (MAR):** Reproduction brought about through various interventions, procedures, surgeries, and technologies to treat different forms of fertility impairment and infertility. These include ovulation induction, ovarian stimulation, ovulation triggering, all ART procedures, uterine transplantation, and intrauterine, intracervical, and intravaginal insemination with semen of husband/partner or donor [90]. **Pharmacodynamics:** Study of pharmacological actions on living systems, including the reactions with and binding to cell constituents, and the biochemical and physiological consequences of these actions [95]. **Pharmacokinetics:** The activity of drugs in the body over a period of time, including the processes via which drugs are absorbed, distributed in the body, localized in the tissues, and excreted [88]. **Quantity:** The physicochemical measure of protein content. If physicochemical tests alone are used to quantitate the biological activity (based on appropriate correlation), potency should be expressed in mass [54]. **Reference standard:** WHO international standards and reference materials provide a common set of standards that are used to ensure the quality of biological medicines worldwide. Currently, the reference standard to measure FSH biopotency is follitropin alfa [93]. **Relative potency:** This is measured by comparing the concentration–response curves of a manufactured test batch with that of a reference standard. **Sialylation:** The covalent addition of sialic acid to the terminal end of oligosaccharides attached to the protein core. Terminal sialylation, namely, the presence or absence of a sialic acid (α2,3 and/or α2,6 N-acetyl neuraminic acid [Neu5Ac]) that caps glycan branches, is the major factor governing in vivo clearance rate. More acidic glycoforms have longer elimination rates due to reduced renal clearance, while less acidic glycoforms have a higher affinity for the FSHR [22]. **Specific activity (units of biological activity per mg of pure protein product):** The quantitative measure of biological activity evaluated as biological activity/mass, and which can also serve to measure the purity and consistency of a product.
